# COVID-19 and the “Film Your Hospital” Conspiracy Theory: Social Network Analysis of Twitter Data

**DOI:** 10.2196/22374

**Published:** 2020-10-05

**Authors:** Wasim Ahmed, Francesc López Seguí, Josep Vidal-Alaball, Matthew S Katz

**Affiliations:** 1 Department of Marketing, Operations and Systems Newcastle University Business School Newcastle University Newcastle Upon Tyne United Kingdom; 2 TIC Salut Social Generalitat de Catalunya Barcelona Spain; 3 Health Promotion in Rural Areas Research Group Gerència Territorial de la Catalunya Central Institut Català de la Salut Sant Fruitós de Bages Spain; 4 Unitat de Suport a la Recerca de la Catalunya Central Fundació Institut Universitari per a la recerca a l'Atenció Primària de Salut Jordi Gol i Gurina Sant Fruitós de Bages Spain; 5 Department of Radiation Medicine Lowell General Hospital Lowell, MA United States

**Keywords:** COVID-19, coronavirus, Twitter, misinformation, fake news, social network analysis, public health, social media

## Abstract

**Background:**

During the COVID-19 pandemic, a number of conspiracy theories have emerged. A popular theory posits that the pandemic is a hoax and suggests that certain hospitals are “empty.” Research has shown that accepting conspiracy theories increases the likelihood that an individual may ignore government advice about social distancing and other public health interventions. Due to the possibility of a second wave and future pandemics, it is important to gain an understanding of the drivers of misinformation and strategies to mitigate it.

**Objective:**

This study set out to evaluate the #FilmYourHospital conspiracy theory on Twitter, attempting to understand the drivers behind it. More specifically, the objectives were to determine which online sources of information were used as evidence to support the theory, the ratio of automated to organic accounts in the network, and what lessons can be learned to mitigate the spread of such a conspiracy theory in the future.

**Methods:**

Twitter data related to the #FilmYourHospital hashtag were retrieved and analyzed using social network analysis across a 7-day period from April 13-20, 2020. The data set consisted of 22,785 tweets and 11,333 Twitter users. The Botometer tool was used to identify accounts with a higher probability of being bots.

**Results:**

The most important drivers of the conspiracy theory are ordinary citizens; one of the most influential accounts is a Brexit supporter. We found that YouTube was the information source most linked to by users. The most retweeted post belonged to a verified Twitter user, indicating that the user may have had more influence on the platform. There was a small number of automated accounts (bots) and deleted accounts within the network.

**Conclusions:**

Hashtags using and sharing conspiracy theories can be targeted in an effort to delegitimize content containing misinformation. Social media organizations need to bolster their efforts to label or remove content that contains misinformation. Public health authorities could enlist the assistance of influencers in spreading antinarrative content.

## Introduction

Since its detection in China in late 2019, SARS-CoV-2 has spread worldwide and been declared a pandemic, with negative effects on both human health and the global economy [1-3]. The dramatic consequences of the pandemic have led to the appearance of numerous conspiracy theories. One of the most successful theories linked 5G to the spread of the disease, leading to misinformation and the burning of 5G towers in the United Kingdom [4]. Other hoaxes have linked the severity of COVID-19 to the genetics of Spaniards and Italians or to vitamin D deficiency [5,6]. There has also been no shortage of hoaxes suggesting that chlorine dioxide can cure the disease [5].

In the first week of April 2020, another conspiracy theory emerged, which suggested that the pandemic was really an elaborate hoax. Drivers of this conspiracy theory argued that it could not exist because hospitals were empty or were operating as normal and therefore there were fewer COVID-19 cases than what had been reported. Its supporters also claimed that the severity of the disease had been exaggerated by the scientific community and the press. This then led to the encouragement of citizens to go to hospitals and film them to show that they were empty. The hashtag #FilmYourHospital was used when posting such videos on social media [7,8].

Misinformation is a matter of public concern. If citizens believe that COVID-19 is a hoax, they may be more eager for lockdown restrictions to ease and they may refuse future vaccines. Recent research has found that those who endorse or believe in conspiracy theories are less likely to adhere to government recommendations such as staying at home or keeping a safe distance between others [9]. The study also found that those who believed in conspiracy theories were also less likely to accept a future diagnostic test or vaccination [9]. In case of future outbreaks, it is important to understand the drivers behind this conspiracy theory so that future ones can be prevented and fought.

In this context, the aim of this study was to analyze the Film Your Hospital conspiracy theory that argued that COVID-19 is a hoax. Specifically, we set out to address the following questions:

1. Who were the drivers of this conspiracy theory on Twitter?
2. What online sources of information were used as evidence to support the theory, including the most retweeted tweets?
3. What was the ratio of automated accounts to organic accounts in the network?
4. What lessons can be learned to mitigate the spread of such a conspiracy theory in the future?

This study is the first empirical investigation into the #FilmYourHospital conspiracy theory and its novelty lies in the social network analysis, identification of influencers, identification of the most shared URLs and hashtags, and identification of genuine accounts as compared to bots. This study may have practical value for the development of recommendations for public health authorities.

## Methods

A computer running Microsoft Windows 8 was used to retrieve data in Microsoft Excel 2010 (Microsoft Corp) using the professional version of the social media analysis software NodeXL (Social Media Research Foundation, release code +1.0.1.428+), which provides access to Twitter’s search application programming interface (API). The study retrieved data from a 7-day period from April 13, 2020, at 14:19 Coordinated Universal Time (UTC) to April 20, 2020, at 15:59 UTC. Users were included in the data set if they sent a tweet during the time the data was retrieved or were mentioned or replied to in these tweets. The keyword FilmYourHospital was used to retrieve data containing mentions of this phrase, including the hashtag #FilmYourHospital. There were 11,333 Twitter users within the network and they generated a total of 22,785 tweets, broken down as follows: 12,905 (56%) retweets, 2736 (12%) replies, 2425 (10.6%) mentions in retweets, 2194 (9.6%) mentions, and 2525 (11%) individual tweets.

Influential users, topics, and web sources were studied, and a social network analysis of the discussion was conducted with NodeXL, using a validated methodology used in previous research [4], which provided an understanding of the shape of the conversation [10-12]. In addition, a network graph was laid out using the Harel-Koren Fast Multiscale layout algorithm [11]. The graph’s vertices were grouped by cluster using the Clauset-Newman-Moore algorithm. NodeXL uses Twitter’s search API. URLs were automatically expanded within NodeXL. The Botometer tool [13] was used to find out the ratio of real to automated/bot accounts being used to tweet about this conspiracy. This was achieved by taking a 10% random sample of users who had sent original tweets and running the sample through Botometer. In understanding the network graph, the results build upon previous seminal research [14] that identified 6 network shapes and structures that Twitter topics may follow [11]: broadcast networks, polarized crowds, brand clusters, tight crowds, community clusters, and support networks. When analyzing popular websites within the network, the content was interpreted by reading the website or watching the video to which the tweet provided a link.

## Results

### Social Network Analysis

**Figure 1:**
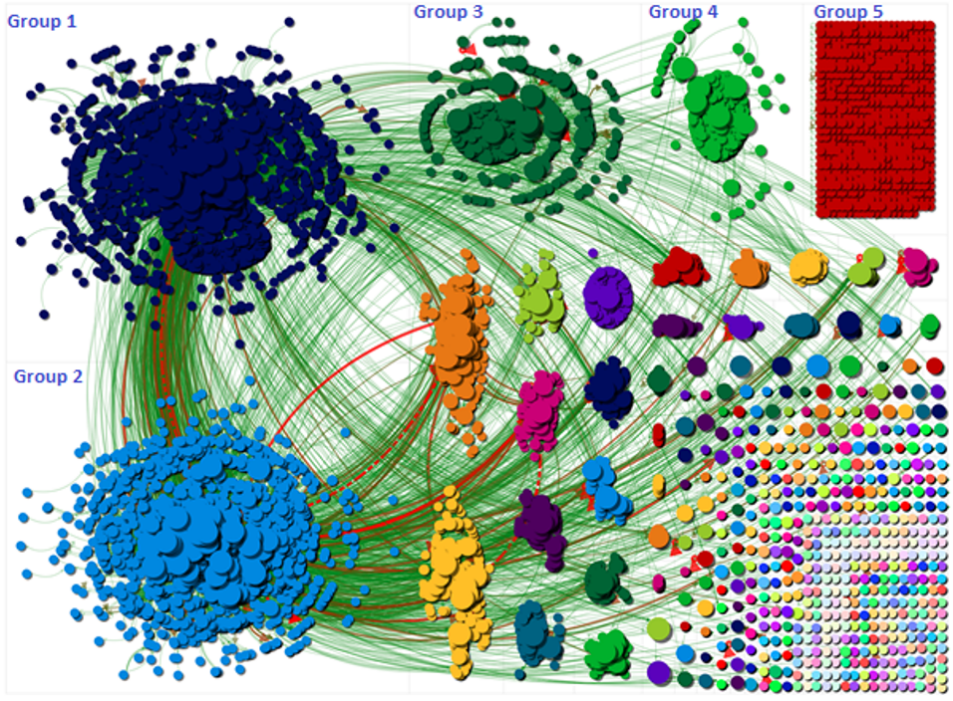
Social network visualization of #FilmYourHospital.

Figure 1 is a social network graph of the discussion surrounding the hashtag #FilmYourHospital. Each small color dot represents a user and a line between them represents an edge. Groups were formed around this topic based on how frequently users mentioned each other. There is an edge for each “replies-to” relationship in a tweet, an edge for each “mentions” relationship in a tweet, and a self-loop edge for each tweet that is not a “replies-to” or “mentions.” The size of the nodes are ranked by their betweenness centrality score (BCS) [14], which measures the influence of a vertex over the flow of information between all other vertices under the assumption that information flows over the shortest paths among them. The graph highlights how a number of different communities tweeting about this topic formed on Twitter. It is important to note that the tweets collected were based on the overall topic and could include a small amount of irrelevant content or users arguing against the conspiracy. The three largest groups (groups 1-3) appeared to be “broadcast groups,” where tweets received a high number of retweets. Group 5 was an “isolates group,” where a number of tweets were sent that did not contain mentions (including retweets). This particular shape appears when a user’s tweet does not contain an @ sign (ie, it is an original tweet expressing a view or opinion that does not refer to any other participant in the discussion). Table 1 provides an overview of the tweets (reworded for anonymity) that contained the most retweets according to the cluster they belonged to within the network.

In groups 1 and 2, we can see that the most retweeted post suggested that the public were being misled about hospitals being empty and that hospitals were not busy or overrun, as had been suggested by the mainstream media. The tweet then called for users to film their hospitals to show this by using the hashtag #FilmYourHospital and for the hashtag to become a trending topic. This tweet was sent by a verified Twitter user. In group 3, the most popular tweet (posted in Spanish) called for users to watch the videos that were being posted on the #FilmYourHospital hashtag and “draw their own conclusions.” In group 4, the most popular tweet drew attention to an article posted by APNews, an American news agency. The tweet that was most popular in the fifth group drew attention to the #filmyourhospital hashtag. Table 2 provides an overview of the URLs that were most shared during this time.

**Table 1:** Most retweeted tweets in groups 1 to 5.

Group	Tweet	Retweet Count
1	*Been to two LA hospitals that are supposed to be jam-packed according to the mainstream media. Yet, they are EMPTY and very quiet! Wonder why they are lying to us. As we can’t trust the news we should begin covering those – please send pictures of your hospital*	21,896
2	*Been to two LA hospitals that are supposed to be jam-packed according to the mainstream media. Yet, they are EMPTY and very quiet! Wonder why they are lying to us. As we can’t trust the news we should begin covering those – please send pictures of your hospital*	21,896
3	*Watch the videos that are being published in the #FilmYourHospital tag and draw your conclusions*	2178
4	*The lame mainstream media has been reporting that the #FilmYourHospital can be traced to Qanon which is a far right group*	671
5	*Come on people – hospitals are empty – only queues are for shopping supplies.* *Corona is a Hoax*	50

**Table 2:** Most shared URLs.

Rank	Title	URL	Count
1	St Louis Doctor has had enough (video removed by YouTube)	https://www.youtube.com/watch?v=shBlwyrBii0&app=desktop	83
2	Health Worker Says EVERYONE Who Dies Has “Corona Virus” on their Death Certificate	https://www.youtube.com/watch?v=ioUREi1myNs&feature=youtu.be	63
3	la tendencia que las redes sociales censuraron (English translation: the trend that social networks censored; video removed by YouTube)	https://www.youtube.com/watch?v=DwY_t1fuuhw&feature=youtu.be	54
4	Polio outbreaks in Africa caused by mutation of strain in vaccine	https://www.theguardian.com/global-development/2019/nov/28/polio-outbreaks-in-four-african-countries-caused-by-mutation-of-strain-in-vaccine	47
5	NHS staff party away in Dudley at Russell Hall Hospital while the world is on lockdown	https://thetattyjournal.org/2020/04/17/nhs-staff-party-away-in-dudley-at-russell-hall-hospital-while-the-world-is-on-lockdown/?fbclid=IwAR2Y9AoddLDdo-K0Sps40xl_MNBiAAzhM1jNpYwRLvTllS5Q59NTWannQoM	25
6	Why are all of these hospital workers dancing in hospitals which appear to be empty?	Removed for anonymity	23
7	Army's Seattle Field Hospital Closes After 3 Days, Without Treating a Single Patient	https://news.yahoo.com/armys-seattle-field-hospital-closes-165646379.html?soc_src=community&soc_trk=ma	20
8	Hospitals have slowed down – yet staff at hospitals are able to spend time making TikToks	Removed for anonymity	19
9	Very peculiar situation in the United States at the moment – also wonder why Twitter felt the need to remove the #FilmYourHospital hashtag	Removed for anonymity	18
10	[User handle removed for anonymity] placed in quarantine until May by filling the hospitals. Yesterday the Municipal Hospital of Tatuapé was empty. It is not to preserve lives, but to break São Paulo.	Removed for anonymity	17

In the first video, it is claimed the person speaking is a doctor, but the comments in the video and the office setting indicate that the person in the video may be a chiropractor. In the second video, a health worker notes that, on mainstream television, COVID-19 has been exaggerated and that all deaths in his hospital are being treated as COVID-19–related. The third most frequent URL shows a person who speaks against the conspiracy campaign and requests that people take the pandemic seriously. The fourth URL notes that polio outbreaks in Africa were caused by the mutation of a strain from a vaccine. The fifth URL linked to a news website critical of National Health Service (NHS) staff. Similarly, the sixth most popular URL linked to a tweet that was critical of medical professionals posting videos of themselves dancing in hospitals on social media. The seventh most popular URL noted that the US army's Seattle Field Hospital closed after 3 days without treating any patients. The eighth most popular URL was a tweet that was critical of medical professionals using TikTok to create videos (the tweet was later deleted). The ninth most shared URL linked to a tweet that questioned why Twitter had removed the trending hashtag #FilmYourHospital. The tenth most shared URL came from a user who claimed that a certain hospital was empty. Table 3 provides insight into the most shared hashtags from this time period that were used alongside the #filmyourhospital hashtag.

The three most frequently used hashtags were #filmyourhospital, #emptyhospitals, and #filmyourhospitals. It was also interesting to see the hashtag #lamestreammedia, which appeared to be critical of the mainstream media. Other hashtags included #endtheshutdown as well as the hashtag #plandemic, which refers to another viral conspiracy theory. Users sharing #endtheshutdown may have tried to use the conspiracy theory as an argument to end the lockdown.

**Table 3:** Most shared hashtags.

Hashtag	Count
filmyourhospital	13,984
emptyhospitals	1160
filmyourhospitals	1144
filmeseuhospital	1045
covid19	840
coronavirus	759
coronahoax	649
lamestreammedia	609
endtheshutdown	478
plandemic	441

### User Analysis

Table 4 ranks influential users who have more than 100 followers by their betweenness centrality score. The followers column notes the number of followers the user had. The in-degree coefficient examines the number of inbound connections and is helpful in highlighting the users who are potentially the most trusted. The out-degree coefficient examines the number of outbound connections by users, which is useful in highlighting the number of accounts users were referring to.

The most popular account belonged to a user who identified as a Brexit supporter, followed by two citizens, one of whom was from Brazil. This user was particularly active during this time and had sent out a total of 154 tweets. A number of accounts (such as 6 and 7) appeared to be in support of the idea that COVID-19 is a hoax and these accounts posted a number of tweets using the #FilmYourHospital hashtag. Account number 6 was the most active and had sent out a total of 163 tweets. The most common type of account in the network belonged to citizens supporting this conspiracy theory. Donald Trump did not tweet using the hashtag; however, he was a trusted node in the network, receiving a total of 310 inbound connections.

**Figure 2:**
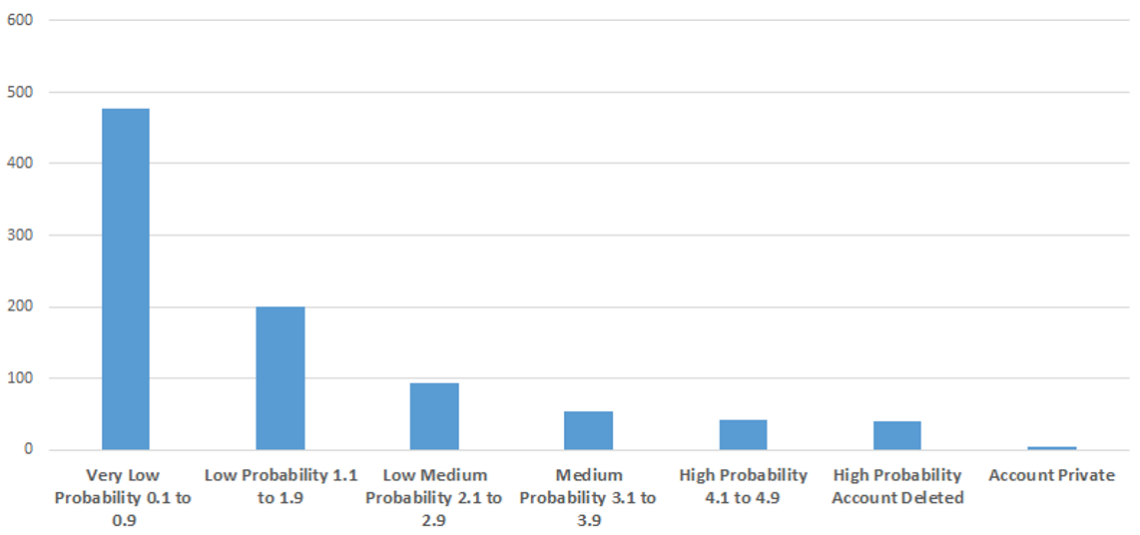
Assessing the number of bots on a scale from 0 to 5 using Botometer.

Figure 2 presents results from Botometer, which was used to assess the frequency of automated accounts. The results indicated that the rate of automated accounts was low (9.2% of the sample), as the majority of accounts had a score of 0.1 to 0.9 (52% of the sample). The accounts scoring 4 or higher were further examined, as were those that had their tweets deleted. It was found that these accounts had sent 136 tweets during the studied time period, although the total combined number of tweets the bots had sent before their accounts were removed was 323,096. The high-probably bot accounts had received a total of 373,508 likes and had a combined total of 22,855 followers. Table 5 highlights the most frequently used words that appeared in the user-bios of users who had a score over 4 and/or whose account was deleted.

It can be seen that high-probability automated accounts and/or deleted accounts contained the words “maga” and “trump” with the highest frequencies within their user bios.

**Table 4:** Influential users.

Rank	User	Betweenness centrality	Followers	In-degree	Out-degree	NodeXL Group in Figure 1
1	Brexit supporter (UK)	14,153,758	1219	7	154	2
2	Citizen	12,085,093	194	695	2	1
3	Citizen	12,055,635	2679	631	2	3
4	President Donald Trump	11,194,004	77,802,730	310	0	2
5	George News	11,138,055	141,840	615	1	4
6	Citizen	7,948,257	191	16	163	2
7	Citizen	6,816,583	5904	0	78	6
8	Citizen	6,107,454	176	239	15	1
9	Nongovernmental organization	4,656,161	14,779	14	130	1
10	Citizen	3,616,519	1838	189	5	2

**Table 5:** Top 5 most frequently used keywords in bios of users who scored 4 or over and whose account was deleted.

Rank	Word	Frequency
1	trump	7
2	maga	7
3	patriot	4
4	support	4
5	love	4

## Discussion

In regard to the first research question, this study found that the most frequent drivers of the #FilmYourHospital conspiracy theory appeared to be ordinary citizens; one of the most influential users is a Brexit supporter. Previous research on other COVID-19 conspiracies has also found that accounts that appeared to belong to citizens were the most influential [11]. Regarding the second research question, we found that YouTube was the information source most linked to by users. The most retweeted post belonged to a verified Twitter user, suggesting that Twitter tools aimed at identifying verified and trustworthy users do not always work. Regarding the third research question, related to automated accounts, we found that there was a low volume of bots in this social network analysis and a low volume of accounts that were deleted within the network.

YouTube was a popular platform for hosting content used to support this conspiracy. Certain tweets sharing the conspiracy theory also became very popular on Twitter. For instance, the tweet sent by the verified user contained a vast number of replies from other users indicating support of the view that COVID-19 is a hoax. Although our results found that some content had been deleted on Twitter and YouTube, there were still tweets and YouTube videos that remained online.

A number of recommendations can be made to attempt to combat the propagation of misinformation. First, as recommended in previous research [15], public health authorities could use the hashtag and enlist the assistance of influencers to share the antinarrative (ie, reasons against the conspiracy). The majority of genuine (versus automated) accounts show the nature and popularity of the conspiracy. Second, bots would need to be countered in a more technical manner. It is important to note that most of them already existed before the beginning of this specific conspiracy theory, suggesting a broader problem. However, not all automated bots are set up for malicious purposes. Social media organizations could monitor for suspicious accounts set up to spread misinformation. Third, as our results show, the “citizen-based” #FilmYourHospital hoax should be countered with untargeted, trustworthy information, delivered from public health authorities as well as popular culture influencers (as mentioned above). Previous academic research suggests that explaining flawed arguments and describing scientific consensus to other people may help lessen the effect of misinformation about science [16]. Lastly, public health authorities and governments should enlist the help of the public in using the “report content” features across social media platforms. With a collective effort in rapidly reporting false and misleading information, a coordinated response would allow it to be detected and removed faster. This tactic may be more effective than the public engaging with the content, which may inadvertently raise its profile.

The network shape of this conspiracy theory differs from that of a conspiracy theory linking 5G and COVID-19, as that theory’s network had a large isolated group where users were tweeting without mentioning each other [11]. Conversely, in the FilmYourHospital network, there appear to be denser groups, indicating a larger volume of retweets and mentions. A reason for this is that certain tweets became very popular, were retweeted with high frequency, and received a large number of replies. This demonstrates that this conspiracy theory was larger in size, attracting more users and tweets: when comparing the 7-day time period of this study to that of the COVID-19 and 5G conspiracy [4], “FilmYourHospital” had almost double the number of tweets. The limitations of this study are that a 7-day time period was examined and Twitter’s search API was used, which may exclude certain tweets. Future research could seek to examine the locations of users that had tweeted using the hashtag and correlate this data with the re-emergence of COVID-19. Another important aspect to note is that there were also tweets that were irrelevant, such as ads, and some tweets might have been from users questioning the conspiracy theory. Not all tweets can definitively be interpreted as supporting the conspiracy. Future research could seek to conduct an analysis of tweets to ascertain the percentage of tweets that were related to the conspiracy. Future research could also seek to analyze the social networks of multiple conspiracy theories and look for potential commonalities with respect to key users and information sources.

## Abbreviations

API: application programming interface
BCS: betweenness centrality score
UTC: Coordinated Universal Time
